# Antioxidant Potential of Sulfated Polysaccharides from *Padina boryana*; Protective Effect against Oxidative Stress in In Vitro and In Vivo Zebrafish Model

**DOI:** 10.3390/md18040212

**Published:** 2020-04-14

**Authors:** Thilina U. Jayawardena, Lei Wang, K. K. Asanka Sanjeewa, Sang In Kang, Jung-Suck Lee, You-Jin Jeon

**Affiliations:** 1 Department of Marine Life Sciences, Jeju National University, Jeju 690-756, Korea; tuduwaka@gmail.com (T.U.J.); comeonleiwang@163.com (L.W.); asanka.sanjeewa001@gmail.com (K.K.A.S.); 2 Marine Science Institute, Jeju National University, Jeju Self-Governing Province 63333, Korea; 3 Department of Seafood and Aquaculture Science, Gyeongsang National University, Tongyeong 53064, Korea; sikang@gnu.ac.kr; 4 Research Center for Industrial Development of Seafood, Gyeongsang National University, Tongyeong 53064, Korea

**Keywords:** antioxidant, Maldives, *Padina boryana*, sulfated polysaccharide, zebrafish

## Abstract

Elevated levels of reactive oxygen species (ROS) damage the internal cell components. *Padina boryana*, a brown alga from the Maldives, was subjected to polysaccharide extraction. The Celluclast enzyme assisted extract (PBE) and ethanol precipitation (PBP) of *P. boryana* were assessed against hydrogen peroxide (H_2_O_2_) induced cell damage and zebra fish models. PBP which contains the majority of sulfated polysaccharides based on fucoidan, showed outstanding extracellular ROS scavenging potential against H_2_O_2_. PBP significantly declined the intracellular ROS levels, and exhibited protection against apoptosis. The study revealed PBPs’ ability to activate the Nrf2/Keap1 signaling pathway, consequently initiating downstream elements such that catalase (CAT) and superoxide dismutase (SOD). Further, ROS levels, lipid peroxidation values in zebrafish studies were declined with the pre-treatment of PBP. Collectively, the results obtained in the study suggest the polysaccharides from *P. boryana* might be a potent source of water soluble natural antioxidants that could be sustainably utilized in the industrial applications.

## 1. Introduction

Aerobic metabolism results reactive oxygen species (ROS) as its by-product. ROS is comprised of both radical and non-radical species. Distinctively, superoxide anion (O_2_^-●^), hydroxyl radical (^●^OH), and hydrogen peroxide (H_2_O_2_) exhibit properties which discuss its involvement in biological targets. ROS possesses two faces; physiological levels support redox biology and pathological levels are explained via oxidative stress. At normal physiological amounts, ROS contributes to the activation of signaling pathways hence initiate biological processes, though oxidative stress damage cellular components including macromolecules such as DNA, lipids, and proteins [1,2]. The effect of ROS elevated levels is counterbalanced with a variety of antioxidants which are divided into two categories namely enzymatic and non-enzymatic. Superoxide dismutase (SOD), catalase (CAT), glutathione peroxidase (GTPx), and glutathione transferase (GST) are foremost components of enzymatic antioxidants. Non-enzymatic antioxidants include compounds with a low molecular weight—such as ascorbic acid (vitamin C), α-tocopherol (vitamin E), glutathione, and β-carotene [3]. However, excessive ROS accumulation makes way to countless pathologies such as inflammation, cancer, and abnormal aging. With regard, supplementary antioxidants are beneficial. Such synthetic antioxidants are butylated hydroxytoluene (BHT), and butylated hydroxyanisole (BHA) [4]. However, given its synthetic nature, the human body is vulnerable to side effects. Thus, research endeavors complying with natural antioxidants from sustainable sources have received much attention.

Seaweeds are a source of bioactive components, capable of producing a myriad of secondary metabolites. Previous literature covers antioxidant, anti-fungal, anti-inflammatory, and anti-tumor potential of compounds from vivid algal species. Though seaweeds undergo harsh environmental conditions, such as high intense light and oxygen concentrations, which support the formation of oxidizing components, they manage to prevail without any serious damage. This fact suggests possession of protective compounds and mechanisms among seaweeds [5,6].

Different species of the genus *Padina* have been subjected to experiments. A range of phytochemicals and their bioactivities were analyzed in *Padina tetrastromatica* [7]. *Padina pavonica* was extensively studied for its sulfated hetero-polysaccharides [8,9]. Inhibition of hyaluronidase activity of the *Padina pavonica* was assessed in its water extract [10].

Polysaccharides are one of the major components of the natural sources available in marine algae. These were reported as effective and non-toxic components with comparatively higher in yield and rather easy to extract, having pharmacological importance [11]. Sulfated polysaccharides inherit distinct attention among other types of polysaccharides due to its numerous bioactivities. Anti-inflammatory [11,12], anti-coagulant [13], anti-proliferative, and antioxidant [14] properties of polysaccharides purified from seaweed species have been studied previously.

This study focuses on the extraction of polysaccharides from brown algae *Padina boryana* collected from the Maldives. Antioxidant potential of the polysaccharides from *P. boryana* has not been reported yet, to the best of our knowledge. Hence, the above properties of the polysaccharides are evaluated in vitro (Vero cells) and in vivo (zebrafish) scale.

## 2. Results

### 2.1. Chemical Composition

The celluclast enzyme assisted extract of *P. boryana* (PBE) and ethanol precipitated component PBP were subjected to chemical composition analysis. The results are given in Table 1. The total phenol content of PBE was 1.32 ± 0.17% while PBP exhibited 1.14 ± 0.26%. Sulfated polysaccharide content was higher in the PBP (56.34%). Monosaccharide analysis revealed, high contents of fucose and galactose in PBP compared to PBE.

**Figure 1:**
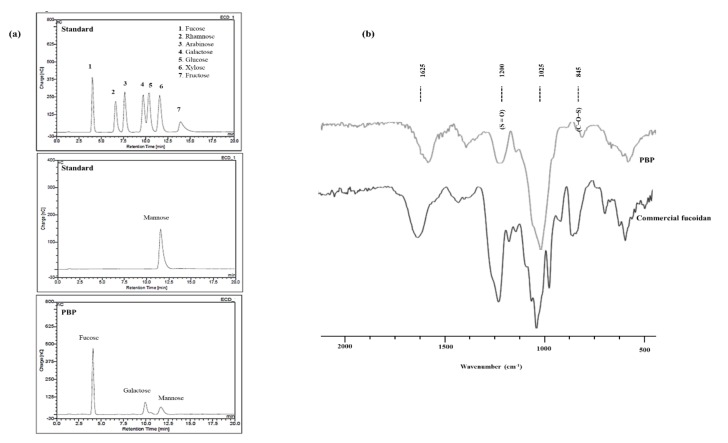
Chemical characterization of PBP. (**a**) Standard monosaccharide and PBP analyzed by HPAE-PAD spectrum. (**b**) ATR-FTIR spectra of PBP and commercial fucoidan.

The analyzed Fourier transform infrared (FTIR) data are illustrated in Figure 1. Glycosidic bonds are formed among multiple monomer units to form polysaccharides and are represented via the 1025 cm^−1^ (C-O-C stretching vibrations) fingerprint peak. An intense peak at the 1200 cm^−1^ is observed due to the sulfate stretching vibrations (S=O). While the bending sulfate vibrations are represented via the absorptions at 845 cm^−1^ (C-O-S). The moisture content available in the sample was observed via the H-O-H bending vibrations in the 1625 cm^−1^ region [12,15,16]. Results suggest the PBP possesses a close correlation with commercial fucoidan.

### 2.2. Free Radical and Hydrogen Peroxided Scavenging Activity

The individual radical scavenging activities corresponding to each sample is expressed in Table 2. Both samples exhibited potent scavenging activities where PBP was more active compared to PBE. Interestingly, H_2_O_2_ chemical assay analysis for scavenging also exhibited PBP as the potent, significant scavenger of radicals. Hence, further experiments were planned with the PBP sample.

### 2.3. Protective Effect of PBP in H_2_O_2_ Stimulated Cells

**Figure 2:**
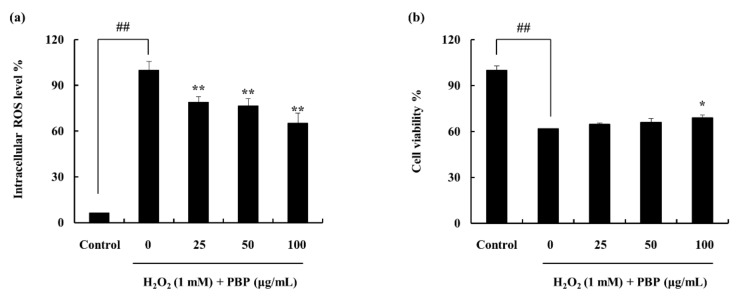
Hydrogen peroxide stimulated Vero cells exhibit oxidative stress. (**a**) Intracellular reactive oxygen species (ROS) scavenging ability of PBP. (**b**) PBPs’ potential to protect cells against hydrogen peroxide. Experiments were triplicated and data shown as mean ± SE; * *p* < 0.05, ** *p* < 0.01. (# denotes significance compared to control while * represents significance compared to H_2_O_2_ treated group).

The PBP exhibited protective effects against the H_2_O_2_ induced cells. The cell viability which was declined with the H_2_O_2_ treatment was reinstated with the PBP treatment. Similarly, the intracellular ROS level which was increased against the H_2_O_2_ stimulation was effectively downregulated with the treatment of PBP. Furthermore, the results exhibited a dose dependent recovery of each indicator (Figure 2).

### 2.4. PBP Protects Cells from H_2_O_2_ Induced Apoptosis

**Figure 3:**
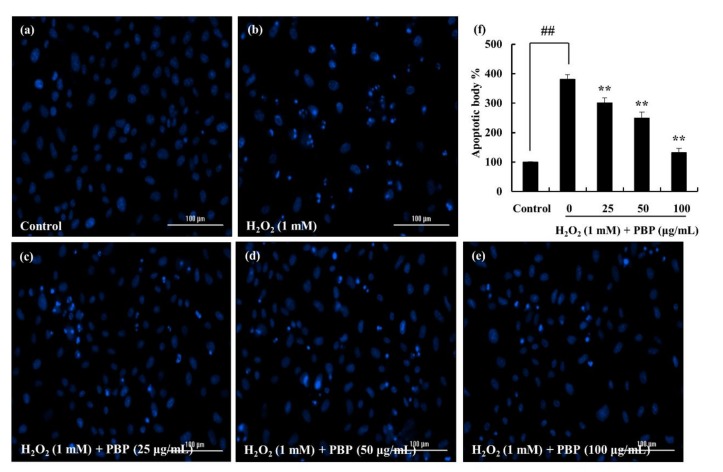
PBP protects Vero cells against H_2_O_2_-induced apoptosis. The apoptotic body formation was observed using Hoechst 33342 staining method under a fluorescence microscope. (**a**) non-treated group, (**b**) H_2_O_2_ treated (1 mM) cells, H_2_O_2_ stimulated cells treated with PBP (**c**) 25 µg/mL, (**d**) 50 µg/mL, (**e**) 100 µg/mL, (**f**) quantitative representation. The intensity levels were analyzed using ImageJ software. Triplicated experiments were conducted and results are represented as mean ± SE; * *p* < 0.05, ** *p* < 0.01. (# denotes significance compared to control while * represents significance compared to H_2_O_2_ treated group).

Earlier studies have revealed the effect of H_2_O_2_ on DNA damage leading to apoptosis [17]. Hence, the effect was evaluated through nuclear staining methods. This particular study followed the Hoechst 33342 staining method. Viable cells are indicated via homogeneously stained nuclei while fragmented and chromatin condensed nuclei are an indication of apoptotic cells [18]. As indicated in Figure 3, cells that were exposed to H_2_O_2_ were associated with increased cell death, indicating higher intensity in the nuclei region. The number of apoptotic bodies was significantly decreased with the PBP treatment, which was indicative of its potential to act as a protective substance against ROS.

### 2.5. Effect of PBP on Antioxidant Enzymes and Pathway Proteins

**Figure 4:**
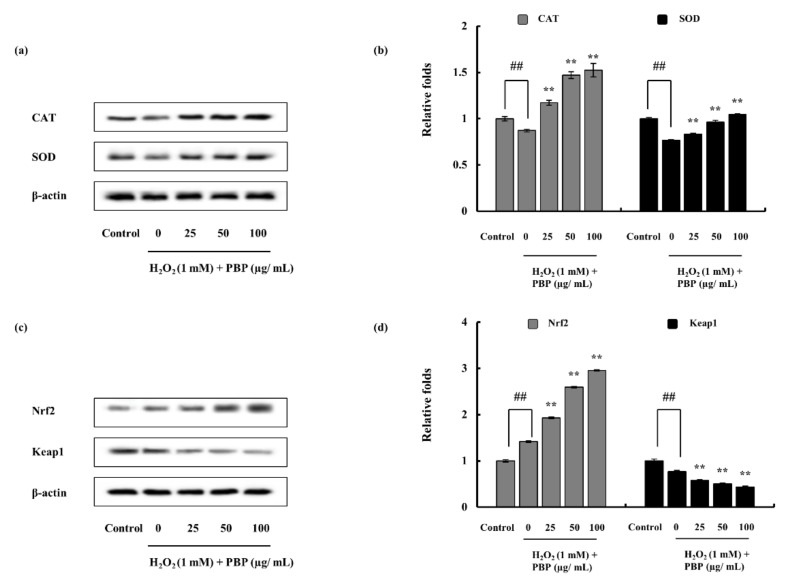
Effect of PBP on the H_2_O_2_ induced antioxidant related protein in Vero cells. (**a**) CAT and SOD, (**b**) relevant quantitative data, (**c**) Nrf2 and Keap1 in cytosol western blot, and (**d**) relevant quantitative data. β-actin was used as internal control. Quantification was assisted with the ImageJ software. Results are represented as mean ± SE; * *p* < 0.05, ** *p* < 0.01. (# denotes significance compared to control while * represents significance compared to H_2_O_2_ treated group).

CAT and SOD are important enzymes in the process of degrading hydrogen peroxide to protect the cells against oxidative damage. It was observed that the particular enzyme protein levels were significantly declined with the H_2_O_2_ treatment. The co-treatment of the PBP recovered the enzyme levels dose dependently overcoming the effect of H_2_O_2_ (Figure 4a,b). Further, the effect was examined in the Nrf2-Keap1 pathway proteins. The cytoplasm nuclear factor E2-related factor 2 (Nrf2) level was increased while Kelch-like ECH-associated protein 1 (Keap1) exhibited declining intensities. It was observed that PBP encouraged the Nrf2 protein expression and stabilized the Keap1 protein allowing successful translocation of Nrf2 to the nucleus (Figure 4c,d). Collectively, the results obtained suggested the potential of PBP to promote Nrf2 expression and nuclear translocation to induce transcription of antioxidant enzymes such as CAT and SOD.

### 2.6. Potential of PBP to Protect H_2_O_2_ Induced Zebrafish in Lipid Peroxidation, ROS Accumulation, and Cell Death

**Figure 5:**
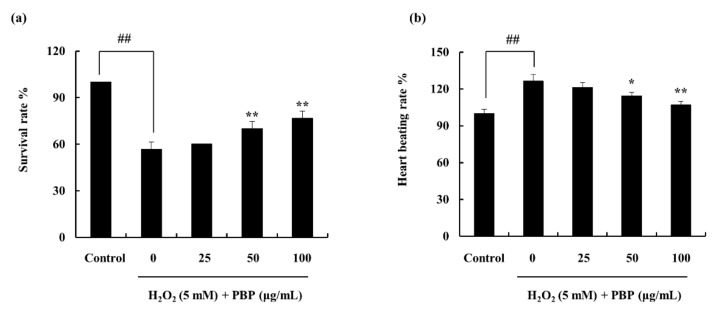
Embryos pre-treated with H_2_O_2_ (5 mM) and followed by PBP treatment (25, 50, 100 µg/mL). (**a**) survival rate, and (**b**) heart beating rate. Experimental procedure followed triplication and data indicated as mean ± SE; * *p* < 0.05, ** *p* < 0.01. (# denotes significance compared to control while * represents significance compared to H_2_O_2_ treated group).

**Figure 6:**
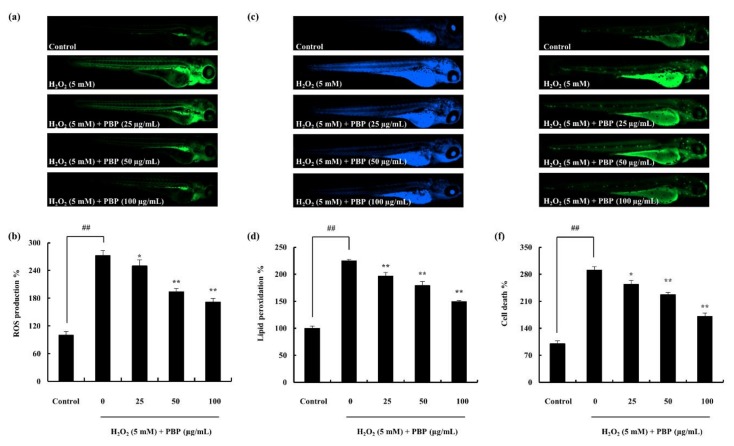
in vivo evaluation of antioxidant potential of PBP. (**a**) Hydrogen peroxide induced ROS production with DCF-DA staining, (**c**) Hydrogen peroxide-induced lipid peroxidation production stained with DPPP, (**e**) PBP protects zebrafish embryos against H_2_O_2_-induced death, stained with acridine orange and captured under fluorescence microscope. Quantitative results of each analysis are represented in (**b**), (**d**), and (**f**) respectively, measured via ImageJ software. Triplicated experiments were conducted and results are represented as mean ± SE; * *p* < 0.05, ** *p* < 0.01. (# denotes significance compared to control while * represents significance compared to H_2_O_2_ treated group).

The survival rate and the heartbeat rate were recorded against the PBP treatment in H_2_O_2_ stimulated zebrafish (Figure 5). The ROS production in the zebrafish embryos treated with H_2_O_2_ was investigated by 2,7-dichlorofluorescein diacetate (DCF-DA). The results are interpreted as an indication of the fluorescence intensity. The H_2_O_2_ treated embryos expressed significantly high fluorescence intensity compared to the non-treated group. PBP pre-treated embryos exhibited a downregulation of fluorescence intensities dose dependently, subduing the effect generated via H_2_O_2_. This reflects the gradual decrement of ROS production, which implies PBPs’ ability to work as a protective agent (Figure 6a,b). The amount of lipid peroxidation was measured using the diphenyl-1-pyrenylphosphine (DPPP) staining. Similarly, the results indicated a decline in the amount of lipid peroxidation with the PBP treatment, (Figure 6c,d). The cell death which was evaluated through the acridine orange staining exhibited the fluorescent intensities to be declined significantly in H_2_O_2_ induced zebrafish embryos with the PBP treatment (Figure 6e,f). The cell death percentage which was 290 in the H_2_O_2_ treated group was declined up to 170 with the PBP (100 µg/mL) treatment. These results reveal the prospective of PBP to act as a potent protector against H_2_O_2_ stimulated oxidative stress.

## 3. Discussion

Marine organisms are reported to be immense sources of secondary metabolites that possess novel biological activities. These sources have proven to be useful in the treatment of diseases and hence be utilized prospectively in pharmacology and medicinal sectors [19]. The antioxidant activity of Hawaiian marine algae has been studied by Kelman et al. (2012), providing an extensive report on different marine algal species including red, brown, and green algae [20]. The sulfated polysaccharides from marine algae as a source of antioxidant secondary metabolite were reviewed by Wijesekara et al. (2011) [21]. Brown algae *Ecklonia cava* was reported as a source of antioxidant components further conducting its abilities in vitro scale. The secondary metabolite of interest; phlorotannin derivatives were reported to be exhibiting noteworthy antioxidant potential [22].

*Padina* is a genus of brown algae in which the thallus is calcified. The seaweed is fan shaped, widely distributed in warm tropical waters from lower intertidal to deep subtidal zones [23]. *Padina boryana*, in particular, has previously been studied by Sanjeewa et al. (2019), purifying fucoidan and evaluating its anti-inflammatory properties [24]. The structure of fucoidan was widely evaluated by Usoltseva et al. (2017) [25]. The species form the Maldives has not been deeply studied for its secondary metabolites specifically against antioxidant potentials. Hence, this study aimed to extract water soluble sulfated polysaccharide from the above under-explored brown algae and to investigate its antioxidant properties, specifically on the ethanol precipitation (PBP) which is rich in fucose.

Polysaccharides among other metabolites receive much attention due to their high availability, and diversified structure with vivid functional groups attached to its backbone [26]. Polysaccharides lack structural homogeneity. Fucoidan is one such polysaccharide containing fucose as its main component. Sulfate groups are substituted in the structure of fucose forming ester bonds. Fucoidan is unique due to its higher content of L-fucose and sulfate groups [27]. Different insertions into the backbone of the structure are also possible (mannose, glucose, and galactose). The point of sulfation and the degree of sulfation could be altered form one species to another. The bioactive properties of fucoidans prevail and increase due to the substitution of sulfate groups. One such report emphasized the action of the anionic sulfate group is that it enhances the nonspecific binding of proteins [28]. Hence, the potential of PBP is attributed to the higher degree of polysaccharide content and its sulfate substitution percentage. Another important component of crude polysaccharide is alginate. The chemical composition indicates higher polysaccharide yield in the PBP and sulfate content exhibits a similar trend. The yield of the polysaccharide is justifiable via the activity of celluclast enzyme to breakdown the cell wall. Furthermore, the dielectric constant of the solution is lowered by the addition of ethanol and polysaccharides are precipitated. The structural characterization of the PBP was supported by the FTIR analysis. The FTIR spectral data collectively revealed the correspondence of PBP to commercial fucoidan.

Previous literature reveals the potential of crude polysaccharides to act as antioxidants. The composition including its functional groups and monosaccharides are reported to synergistically induce the free radical scavenging activity [29,30]. Therefore, monosaccharide composition of the samples was analyzed and results indicate an increment in fucose and galactose contents. Higher values of fucose and galactose composition were available in PBP compared to PBE accrediting PBPs’ elevated potential to act as an antioxidant.

DCF-DA assay was used in the evaluation of intracellular ROS scavenging activities. The stain is absorbed into the cells through the membrane and converted to DCFH, a non-fluorescent component, via the cellular esterases. Thus, intracellular ROS converts DCFH to fluorescent active DCF and detectable with a fluorimeter. Intracellular ROS levels were observed to be upregulated with H_2_O_2_ pre-treatment while treatment with PBP dose-dependently downregulated it. Furthermore, the protective effects of PBP against H_2_O_2_ were observed by cell viability analysis.

Oxidative stress is caused due to the presence of reactive oxygen species (ROS). Cellular metabolism and environmental factors contribute to the production of ROS. At moderate concentrations, ROS plays an important role in the function of physiological cell processes while high concentrations lead to adverse effects [31]. The highly reactive molecules damage cell structures including macromolecules thus alter the physiological functions. Moreover, the imbalance between the ROS and its counterpart antioxidants creates oxidative stress. Cell viability, proliferation rate, and further functions are affected by oxidative stress. Even though aerobic organisms possess integrated antioxidant systems, under pathological conditions these systems can be overwhelmed [3]. H_2_O_2_ is a distinct ROS species with physiological significance among others such as superoxide anion (O_2_^-●^) and hydroxyl radical (^●^OH). H_2_O_2_ is produced in the cells upon phagocytosis due to the superoxide burst and converted. Xanthine oxidase, NAD(P)H oxidase, and amino acid oxidase contribute in the production of hydrogen peroxide [32]. In the presence of transition metal ions, H_2_O_2_ break down and result in OH^-^ and ^●^OH via the Fenton reaction [33]. Therefore, this study focuses on the stimulation via H_2_O_2_ under both in vivo and in vitro conditions.

The genetic material is modified by the ROS via different mechanisms involving DNA strand breakage, sugar moiety modifications, base unit degradation, deletions, and translocations [34]. Possible DNA modifications lead to carcinogenesis, aging, and numerous diseases. The effect was evaluated in the study via nuclear staining methods. It was evident the effect of H_2_O_2_ on the condensation and fragmentation of the nuclear material which was dose dependent and down regulated via the treatment of PBP.

Superoxide anion radicals are scavenged via SOD, while CAT plays an important role in the detoxification of the H_2_O_2_. Hence, these could eliminate free radicals and help the conversion of reactive toxic components to non-toxic elements and protect the live organelles from oxidative damage. The antioxidant enzymes were initially hampered by the excess ROS created via the effect of H_2_O_2_, though the potential of PBP recovered the enzyme expression hence reducing ROS production. Keap1 is an inhibitor protein, a cysteine-rich protein that is anchored to the actin cytoskeleton. It is responsible for the cytosolic sequestration of Nrf2 under physiological conditions. Keap1 promotes ubiquitination and degradation of Nrf2 under normal physiological conditions. Under stressful conditions in which the Nrf2-dependent cellular mechanism is active (electrophiles and oxidants are rich in this stage), the Nrf2 is rapidly released from Keap1. Dissociated Nrf2 is translocated to the nucleus and binds to antioxidant response element (ARE). Keap1 also receives redox information or environmental cues via its highly reactive cysteine residues and referred to as the sensor of the Nrf2-Keap1 system [35]. The dissociation of the system is a relatively rapid event. The breakdown of the system leads to Keap1 stabilization. Nrf2 also increases its half-life [36]. This allows successful nuclear translocation and cytoprotective gene transcription. A similar effect of antioxidant polysaccharides was reported by Zhou et al. (2019) [37].

The membrane lipid bilayer is disrupted via ROS stimulated lipid peroxidation thus membrane bound receptor activities are altered leading to increased tissue permeability [38]. Lipid peroxidation results in unsaturated aldehydes which are potent inactivators of cellular proteins through the formation of cross linkages [3]. The effect of ROS was evaluated in vivo scale using zebrafish. The ROS production levels examined via DCFDA fluorescent staining initially indicated the decline of intensities revealing the protective effect of PBP. Furthermore, DPPP staining exhibited the lipid peroxidation intensities to be upregulated under H_2_O_2_ stimulation and significant decline under PBP pre-treatment. The results confirmed the antioxidant potential of PBP against H_2_O_2_ induced oxidative stress. Thus, zebrafish cell death was successfully downregulated dose-dependently as indicated. The results obtained in the study well aligns with an earlier report on polysaccharide extracts from *Hizikia fusiforme* [39]. Furthermore, fucoidan purified from brown algae has incorporated zebrafish studies as an in vivo model [12,40].

Zebrafish involvement in developmental biology and drug discovery has been recognized and the implementation was advanced throughout the years. The size, husbandry, and early morphology is a distinct advantage of the usage of zebrafish over other vertebrate species. This provides researchers to minimize the costs in maintenance as well as in quantities of dosing samples, further in histological assessments [41]. High fertility including transparent embryos makes the species a valuable source [42]. Fluorescent staining methods implemented in the present study also support the above fact.

## 4. Material and Methods

### 4.1. Materials

African monkey kidney cell line (Vero) was purchased from the Korean cell line bank (KCLB, Seoul, Korea). Media for the cell line maintenance (Roswell Park Memorial Institute-1640; RPMI-1640) and serum (fetal bovine serum; FBS) including antibiotics (penicillin, streptomycin) were purchased from Gibco-BRL (Grand Island, NY, USA). 3-(4,5-dimethylthiazol-2-yl)-2,5-diphenyltetrazolium bromide (MTT), 1-diphenyl-2-picrylhydrazyl (DPPH), 2,2-azobis(2-amidinopropane) hydrochloride (AAPH), 5,5-dimethyl-1-pyrolin N-oxide (DMPO), and α-(4-Pyridyl-1-oxide)-N-tert-butylnitrone (POBN) were purchased from Sigma (St. Louis, MO, USA). H_2_O_2_ and FeSO_4_.7H_2_O used were purchased from Fluka Co. (Buchs, Switzerland). Furthermore, dimethyl sulfoxide (DMSO), 2,7-dichlorofluorescein diacetate (DCF-DA), and 2,2′-azino-bis(3-ethylbenzthiazoline)-6-sulfonic acid (ABTS) were obtained from Sigma-Aldrich (St. Louis, MO, USA).

### 4.2. Collection of Seaweed and Extraction

*P. boryana* samples were collected from the shores of Fulhadhoo Island (4.8849 °N, 72.9350 °E), the Maldives in January of 2018. Samples were immediately washed with running water to remove epiphytes and sand. Samples were then lyophilized and ground into powder. A sample portion (50 g) was added to 500 mL of distilled water. Optimal pH value was obtained via the addition of 1M of HCL. Celluclast assisted extraction was carried out in a period of 24 h under shaking kinetics with optimal conditions (pH 4.5, 50 °C). Following the extraction, the enzyme was inactivated via heating the mixture in 100 °C for 10 min. The filtrate was obtained and pH was brought back to neutral value. The sample was identified as enzymatic extract of *P. boryana* (PBE).

### 4.3. Crude Polysaccharide Preparation

The above enzymatic extract was mixed with 95% ethanol (1:3) and was maintained in 4 °C for >8 h period. The precipitate was polysaccharide from *P. boryana* and was designated as PBP.

### 4.4. Chemical Analysis

The chemical composition of both the PBE and PBP was analyzed using several methods. Official methods of analysis of the Association of Official Analytical Chemists (AOAC) was used to obtain the total polysaccharide content [43]. The polyphenol content was measured accordingly with the method described by Chandler and Dodds (1983) with minor modifications [44]. The sulfate content was evaluated by BaCl_2_ gelation method [45].

Samples were hydrolyzed in 4M triflouroacetic acid (4 h, 100 °C). This was subjected to CarboPac PA1 cartridge column (4.5 × 50 mm) for separation and detected with an ED50 Dionex electrochemical detector (Dionex) concerning monosugar analysis [46].

The attenuated total reflectance Fourier transform infra-red (ATR-FTIR) spectrum was obtained with a Bruker FTIR, Alpha II (Bruker, Karlsruhe, Germany) instrument in the 400–4000 cm^−1^ wavenumber range. Commercial grade fucoidan was analyzed at the same time.

### 4.5. Radical Scavenging Activity Evaluation via Electron Spin Resonance (ESR) Spectrometer

Both PBE and PBP were analyzed for its radical scavenging activities. DPPH, alkyl, and hydroxyl radical scavenging activities were evaluated using electron spin resonance spectroscopy (ESR, JES-FA200; JEOL, Tokyo, Japan). The DPPH radical scavenging was assessed via the method defined by Nanjo et al. (1996) [47]. The method, in brief, equal volumes of sample and DPPH was mixed vigorously, transferred to a capillary tube and inserted to the ESR spectrometer for measurement. Alkyl radical scavenging followed the method described by Hiramoto et al. (1993) [48]. The radical was generated via a reaction mixture of AAPH and 4-POBN with tested sample which was incubated in water bath (37 °C, 30 min) and subjected to analysis. The method explained by Finkelstein et al. (1980) was used in the evaluation of the hydroxyl radical scavenging potential [49]. This used the Fenton reaction as the basis and mixed sample in phosphate buffer solution (pH 7.4) with equal volumes of 0.3 M DMPO, 10 mM FeSO_4_, and 10 mM H_2_O_2_ (200 µL) for analysis.

### 4.6. Chemical Assay for Hydrogen Peroxide

A colorimetric assay described by Kim et al. (2014) was implemented in the evaluation of the hydrogen peroxide scavenging [50]. The method in brief; each sample was mixed with 0.1 M phosphate buffer (pH 5.0, 100 µL) in a micro well plate. Hydrogen peroxide (20 µL) was added and was incubated (37 °C, 5 min). ABTS (1.25 mM, 30 µL) and peroxidase (1 unit/mL, 30 µL) was added to the above and further incubated at 37 °C for 10 min. The absorbance measurements were collected using an ELISA reader at 405 nm.

### 4.7. Protective Effects of PBP via In Vitro Methods

#### 4.7.1. Cell Culture

RPMI-1640 medium supplemented with heat-inactivated FBS and antibiotics (penicillin and streptomycin) was used to culture the Vero cells. The cells were maintained in controlled environment (humidified, 5% CO_2_). Periodic subculture was continued and cells were subjected to experiments at its exponential growth phase.

#### 4.7.2. Cell Viability and Intracellular ROS Scavenging Activity in H_2_O_2_ Stimulated Vero Cells

Cells were seeded (1 × 10^5^ cells/mL) and were incubated for 16 h, samples were treated and incubated for 1 h. Following the cells were stimulated with H_2_O_2_ (1 mM). The cell viability was measured given 24 h incubation time using the MTT assay [51]. The intracellular ROS scavenging potential of the samples was measured using the dichloro- fluorescein diacetate (DCF-DA) assay [52]. Initially, the cells were seeded (1 × 10^5^ cells/mL), incubated and treated with different sample concentrations. Cells were stimulated with H_2_O_2_ given 1 h incubation time and DCF-DA (500 µg/mL, stock) was treated to each well. The results were detected as a fluorescence measurement (Ex-485 nm, Em-530 nm) with a microplate reader (BioTech, Winooski, VT, USA).

#### 4.7.3. H_2_O_2_ Induced Cell Apoptosis through Nuclear Staining

The cells were seeded as explained above, treated with samples and was induced with H_2_O_2_. Following a 24 h incubation period, the cells were stained with cell permeable DNA dye Hoechst 33342 (10 µg/mL). Given 10 min incubation period, the cells were observed by a fluorescence microscope equipped with a CoolSNAP-Pro color digital camera (Olympus, Tokyo, Japan) [53,54].

#### 4.7.4. Western Blot Analysis

Protein expression levels of catalase (CAT), superoxide dismutase (SOD), nuclear factor E2-related factor 2 (Nrf2), and Kelch-like ECH-associated protein 1 (Keap1) were analyzed via western blotting. Cells were seeded in 6 well culture plates and samples were treated given 24 h period, following 1 h incubation cells were stimulated with H_2_O_2_. Cells were harvested after complete incubation and lysed. Protein content was measured and standardized. Following electrophoresis, it was transferred on to nitrocellulose membranes. Blocked membranes (5% skim milk) were incubated with primary and secondary antibodies step wisely (Santa Cruz Biotechnology, Paso Robles, CA, USA). The bands were developed and photographed via a FUSION SOLO Vilber Lourmat system. ImageJ program was assisted in the quantification of the band intensities [55,56].

### 4.8. In Vivo Antioxidant Effects of PBP Using Zebrafish Model

#### 4.8.1. Zebrafish Maintenance

Zebrafish in their adult stage were purchased from Seoul Aquarium, Korea. The fish were maintained in acrylic tanks under controlled conditions (28.5 °C, with a 14/10 h light/dark cycle). Fish were fed with tetramin flake, including live brine shrimp, three times per day in equal intervals for 6 days of the week. Natural spawning was stimulated with lights on conditions to obtain the embryos and completed collection within 30 min.

#### 4.8.2. Polysaccharide Application to Zebrafish Embryos

The embryos were transferred to 12 well plates after 7–9 h post-fertilization (hpf). The embryos were maintained in an embryo medium. Samples were treated and incubated for 1 h and stimulated with H_2_O_2_ (5 mM) and continued incubation for 24 hpf. The live embryos were counted after 3 days of post-fertilization (dpf) to obtain the survival rate.

#### 4.8.3. Intracellular ROS, Lipid Peroxidation, and Viability Analysis

At 2 days of post fertilization (dpf), the heartbeat rate was evaluated. Both the atrium and ventricle heartbeat rate was assessed under microscope for 1 min. The zebrafish were initially treated with sample and was induced with H_2_O_2_ [57].

DCF-DA was used to detect the intracellular ROS levels in the zebrafish embryos while lipid peroxidation was assessed via DPPP. Moreover, cell death was evaluated with acridine orange staining. Following each staining method, the embryos, zebrafish larvae were rinsed with embryo media and anaesthetized with 2-phenox ethanol. A microscope equipped with CoolSNAP-Pro color digital camera (Olympus, Tokyo, Japan) was assisted in observation and photography. The intensity quantification was completed with the ImageJ program [58].

### 4.9. Statistical Analysis

The experiments were triplicated and expressed data as the mean ± standard error (SE). One-way ANOVA was implemented in the comparison of mean values. Significance among the treatments were evaluated by Student’s *t*-test (*p* < 0.05, *p* < 0.01).

## 5. Conclusions

This study evaluated sulfated polysaccharide from marine brown alga *P. boryana* ethanol precipitation (PBP) as a source of natural antioxidants. Preliminary chemical characterization revealed its composition and the contribution of sulfate content, fucose, and galactose towards the bioactive properties. Results suggest the ability of PBP to protect ROS mediated cell damage and to inhibit oxidative stress in zebrafish. The increased antioxidant pathway protein expression of Nrf2 and its resulting CAT, SOD protein levels accompanied the effect of PBP. Hence, PBP is a potential source of antioxidants that could be successfully utilized in healthy functional and cosmeceutical sectors.

## Figures and Tables

**Table 1:** Chemical composition of PBE and PBP obtained from *P. boryana.*

Sample		PBE	PBP
Polysaccharides content %		42.14 ± 0.86	49.36 ± 0.79
Sulfates content %		4.57 ± 0.64	6.98 ± 0.35
Phenolic content %		1.32 ± 0.17	1.14 ± 0.26
Mono sugars %	Fucose	39.84	57.51
	Galactose	15.11	21.35
	Mannose	18.24	13.21
	other	24.81	5.63

**Table 2:** Free radical/ROS scavenging activities of PBE and PBP.

	Free Radical/ROS Scavenging Activity (IC_50_, mg/mL)			
	DPPH	Alkyl	Hydroxyl	H_2_O_2_
PBE	4.26 ± 0.14	3.88 ± 0.13	1.96 ± 0.17	1.17 ± 0.11
PBP	3.66 ± 0.44	2.87 ± 0.07	1.06 ± 0.21	0.58 ± 0.04

All results expressed as means ± SE, based on triplicated trials.
